# Determination of the Mutagenicity Potential of Dillsun Herbal Medicine by Single Cell Gel Electrophoresis in Rat Hepatocytes

**Published:** 2013-05-04

**Authors:** Heibatullah Kalantari, Mohsen Rezaei, Maryam Salehcheh, Mehrnoosh Moosavi, Golnaz Varnaseri

**Affiliations:** 1 Department of Pharmacology and Toxicology, Medicinal Plants and Natural Products Research Center, School of Pharmacy, Jundishapur University of Medical Sciences, Ahvaz, IR Iran

**Keywords:** Toxicity Tests, Comet Assay, Herbal Medicine

## Abstract

**Background:**

Traditional medicines are among the oldest medicines and their extensive use in the recent years reflects the public’s interest in alternatives to conventional medicine.

**Objectives:**

The aim of this study was to investigate the genotoxicity of Dillsun herbal medicine in DNA damage of rat hepatocytes compared to sodium dichromate using a comet assay technique.

**Materials and Methods:**

Male Wistar rats were caught and their liver was washed with a perfusion buffer, followed by another wash with collagenase buffer. Hepatocytes were isolated and transferred on to a petri dish which contained a washing buffer. Hepatocytes were then separated and the cells were filtered and centrifuged at 1500 rpm for 3 minutes. The hepatocytes were counted using neubauer slides and kept in a bioreactor for 30 minutes. Cells were then exposed to different doses of Dillsun such 0.2, 1, 2.5, 5 and 10 mg/mL. Sodium dichromate was the positive control and incubated buffer was used as a negative control. Cell suspensions were placed on slides pre-coated with low melting point agarose and were covered with agarose gel. Agarose gels were then lysed and electrophoresis was done, followed by neutralization and staining. Slides were analyzed by fluorescence microscopy. The size and extent of DNA damage visualized by this technique was evaluated by examining cells. Migration behavior was classified according to the Kobayashi pattern.

**Results:**

The results indicated that with an increase of Dillsun dose, the mutagenicity index slightly increased but compared to the positive control, there were significant differences, which suggests that the crude extract of Dillsun in vitro did not have mutagenic effects.

**Conclusions:**

In conclusion the results showed that Dillsun has no mutagenic effects when compared to the positive control. Although by increasing the Dillsun dose, DNA damage also increased but this increase was not significant.

## 1. Background

Human’s use of plants as medicines has a history that goes back almost 60000 years. Herbs were used as medicines for healthcare in every culture throughout history. Neanderthals understood the benefits of herbs and used them as a source of food, clothing, shelter and medicine, and protected them from wild animals and natural hazards (1). Nowadays people throughout the world use plants as an integral part of primary health care (2). Researchers have validated many traditional remedies throughout the world (3), and have seeked information about isolation of bioactive compounds of plants to use them directly in medicines (4). The pharmacopeia of medicines has been set according to herbal knowledge of indigenous people. This information was slowly completed and became the basis of traditional medicine all over the world. In some communities use of herbal medicine is a key part of the medical system (1). Information about the adverse effects of used plants has not been proven in articles. Long-term use of traditional medicine can cause toxicity in humans and can be dangerous for health care. However, recent studies have showen that the herbs used in food or in medicine can be mutagenic substances (5) and the long-term usage of traditional herbs has potential mutagenic risks (6). Recently, single-cell gel electrophoresis (SCGE) assay, also known as the comet assay has become the new technique of choice because this method is rapid and sensitive and can detect various DNA damages (strand breaks and alkali labile sites) in cells, and can be induced by genotoxic agents (7). Cells are embedded in a thin layer of agarose on a microscope slide and lysed with detergent and a high salt solution (8). In Iran, Dill (Anethum graveolens) is a widely used herbal remedy. Dillsun is a pharmaceutical preparation in the form of an oral drop originating from Anethum graveolens which is widely used as a diuretic and anti-spasmodic for gastrointestinal disorders and also as a lipid lowering agent in therapy (9). Each mL of this product contains 20 mg of Anethum graveolens essence. Therefore, one of the objectives of the current study was to evaluate the safe activity of Dillsun as herbal medicine by an in vitro experiment on hepatocytes. The liver is the major organ involved in carcinogen activation and isolated hepatocytes represent the most appropriate in vitro model to mimic the activation profile. The assessment of metabolism activation is of particular importance in the interpretation of the results of mutagenicity studies as many carcinogens are not active per se but require metabolic activation to form the ultimate carcinogens. Studies using the comet assay frequently focus on the liver as the site of metabolism (10). In recent years, the biological studies of herbal medicine using in vitro methods have changed with one the advancements being the development of the comet assay. This technique shows the ratio of living cells to total cells in the cell culture. This method is rapid and affordable and is used for evaluating the mutagenicity or genotoxicity of pharmaceutical compounds (11, 12).

## 2. Objectives

Given the therapeutic use of Dillsun and the lack of information on the genotoxicity of this drug in eukaryotic cells, it appears that the study of probability and improbability of DNA damage in rat hepatocytes by the comet assay, induced with Dillsun must be evaluated.

## 3. Materials and Methods

Dimethyl sulfoxide (DMSO), ethidium bromide, NaCl, KCl, MgSO_4_, Na_2_SO_4_, KH_2_PO_4_, Na_2_HPO_4_, NaHCO_3_, CaCl_2_, hydroxy ethyl piperazine ethane sulfonic acid (HEPES), bovine serum albumin (BSA), collagenase, Na_2_Cr_2_O_7_, NaOH and Di sodium EDTA, were purchased from Sigma (USA), ethylene glycol bis tetra acetic Acid (EGTA), tris, sodium lauryl sarcosinate (C_15_H_28_NO_3_NA) and Triton X-100 were purchased from Merck (Germany), low melting point agar (LMPA) and normal melting point agar (NMPA) were purchased from Fermented (EU) and Dillsun was purchased from barigessence Co (Iran). Hepatocytes extraction buffers include: 1-Hanks × 10 buffer: composed of NaCl (40 g), KCl (2 g), MgSO_4_. 7 H_2_O (1 g), Na_2_ HPO_4_ (0.3 g), KH_2_PO_4_ (0.3 g) and dH_2_O q.s to 500 mL. 2- Hanks I buffer: composed of HEPES (1.5 g), NaHCO_3_ (1.05 g), Hanks × 10 (50 mL) and dH_2_O q.s to 500 mL. 3-Krebs buffers: composed of NaCl (6.95 g), KCl (0.355 g), KH_2_ PO_4_ (0.16 g), MgSO_4_ (0.295 g), CaCl_2_ (0.287 g), NaHCO_3_ (2.1 g) and dH_2_O q.s to 1000 mL. 4- perfusion buffer: composed of BSA (1.3 g), EGTA (0.039 g) and Hanks buffer q.s to 200 mL. 5- collagenase buffer: composed of collagenase (0.065 g), CaCl_2_ (0.03 g) and Hanks I buffer q.s to 100 mL. 6-washing buffers; composed of BSA (2 g), HEPES (0.06 g) and Krebs buffer q.s to 200 mL. 7- incubation buffer: composed of HEPES (0.75 g) and Krebs buffer q.s to 250 mL. Comet assay buffers include: 1-lysis buffer: composed of NaCl (14.61 g), Na_2_EDTA (3.72 g), Tris (0.125 g), Sodium lauryl sarcosinate 1% (1g), NaOH (0.9 g), DMSO%10 (10 mL), Triton X-100 1% (1 mL) and dH_2_O q.s to 100 mL. 2-electrophoresis buffer: composed of NaOH (12 g), Na2EDTA (0.372 g) and dH_2_O q.s to 1000 mL. 3-neutralization buffer: composed of Tris (12.1 g) and dH_2_O q.s to 250 mL. Male Wistar rats weighing 230 – 270 g were obtained from Razi Institute animal house (Iran) and housed in a polyethylene cages with a 12 h light / dark cycle and were fed ad libitum. Animals were anaesthetised by intraperitoneal injection of ketamine (90 mg/kg) and xylazine (10 mg/kg). The rats were then dissected by IV injection of heparin and canulation of liver was made. Liver was perfused through the portal vein, first with perfusion buffer for 10 minutes and then with collagenase buffer for 12 minutes at a flow rate of 25 mL/min. Cells were mechanically separated and the digested liver was filtered to obtain a cellular suspension; the cell suspension was centrifuged for 3 minutes at 1500 rpm. The upper layer was discarded and 10 mL of washing buffer was added to the lower portion and was well mixed. Cell viability was determined by the trypan blue test. From the cell suspension 100 μL was mixed with 200 μL of incubation buffer and 300 μL of trypan blue. Then a drop of cell suspension was placed on neubauer lam and the cells were counted. Live cell and dead cell numbers were as follows: The percentage of viable cells = (average of live cells/total cells) × 100. 10 mL of the cell suspension was put in a bioreactor bath in an atmosphere of 10% O_2_, 85% N_2_ and 5% CO_2_ at 37 ^°^C for 30 minutes and was then poured in to five bioreactor flasks that contained hepatocytes. Five doses of Dillsun, including 0.2, 1, 2.5, 5 and 10 mg/mL (test groups) were prepared and hepatocytes were exposed to different doses of Dillsun for 60 and 120 minutes. Incubation Buffer and sodium dichromate were used as negative and positive controls respectively. The slides covered with normal agarose were prepared and were placed at room temperature to dry. 1 mL of cell suspension from each flask was taken and diluted with 10 mL of low melting point agarose. 100 μL of this suspension was layered on slides, and cover-slips were fixed on the slides. Afterwards they were put in the freezer for 10 minutes, then they were removed from the freezer and the cover-slip was displaced. They were placed in the lysis buffer for 1 hour and washed in deionized water at completion, then they were placed in electrophoresis buffer and electrophoresis was done for 20 minutes at 25 V. After electrophoresis, slides were neutralized with neutralizing buffer and washed three times by this buffer each time for five minutes. DNA was stained with ethidium bromide (2 μg/mL) for five minutes before microscopic evaluation of the DNA damage, based on the method described by Speit and Hartmann (13), which is according to the original work of Singh et al. (14). Slides were evaluated by fluorescence microscopy. The amount of DNA damage was analyzed by the comet assay. The parameters used for statistical analysis were the tail moment and tail length (distance from the middle of nucleoid core to the end of the tail). Cells were visually scored into comet classes according to their tail characteristics: Class 0 = there was no tail, Class 1 = the tail was shorter than the diameter of the head (nucleus), Class 2 = the tail was 1 to 2x the diameter of the nucleus and Class 3 = the tail was longer than 2x the diameter of the nucleus (15-17). Lack of nucleus in DNA or very wide tailed DNA was deleted from this study because it could be a cell (18, 19). Tail length and mutagenic index were calculated with the following formula MI = (0 NMC + 1 SMC + 2 MMC + 3 LMC) /200, that could be explained as NMC = no migration cells (score 0), SMC = short migration cells (score 1), MMC = medium migration cells (score 2), LMC = long migration cells (score 3).

## 4. Results

The percentage of rat hepatocytes comet pattern at different concentrations of Dillsun and their comparison with the positive control (sodium dichromate) at 60 and 120 minutes after exposure displayed significant differences (P < 0.001) as shown in Table 1. The percentage of mutagenic index (MI) and damaged cells that were exposed to different doses of Dillsun at 60 and 120 minutes and their comparison with the positive control (sodium dichromate) which indicated significant differences P < 0.001, are shown in Table 2.

**Table 1.** The Percentage of Rat Hepatocytes Comet Pattern Frequency at Different Doses of Dillsun Herbal Medicine and thier Comparison With Positive Control (Sodium Dichromate) at 60 and 120 Minutes After Exposure Which Indicates Significant Differences P < 0.0001

Substance	Score 0, Mean ± SD	Score 1, Mean ± SD	Score 2, Mean ± SD	Score 3, Mean ± SD
**Sodium Dichromate**				
1 h	8.5 ± 1.21	16± 1.3	28.5 ± 1.3	0 ± 0
2 h	-	-	-	0 ± 0
**Dillsun 0, µL/mL**				
1 h	94 ± 1.62	5.5 ± 1.09	0.5 ±0.57	0 ± 0
2 h	94 ± 0.93	5 ± 0.65	1 ±0.65	0 ± 0
**Dillsun 10, µL/mL**				
1 h	94 ± 1.62	5.5 ±1.09	0.5 ± 0.57	0 ± 0
2 h	94 ±0.65	5 ± 0.65	1 ± 0.65	0 ± 0
**Dillsun 50, µL/mL**				
1 h	93 ±1.09	6 ± 1.3	1 ± 1.09	0 ± 0
2 h	92 ± 1.48	6.5 ± 1.44	1.5 ± 0.57	0 ± 0
**Dillsun 125, µL/mL**				
1 h	91 ± 1.09	7.5 ± 0.93	1.5 ± 1.09	0 ± 0
2 h	90 ± 1.09	8 ± 1.09	2 ± 0	0 ± 0
**Dillsun 250, µL/mL**				
1 h	90 ± 1.44	8 ± 1.09	2 ± 0.65	0 ± 0
2 h	89 ± 1.09	8 ± 1.48	3 ± 0.57	0 ± 0
**Dillsun 500, µL/mL**				
1 h	88 ± 1.09	9 ± 0.57	3 ± 0.93	0 ± 0
2 h	87.5 ± 1.48	9 ± 1.32	3.5 ± 0.87	0 ± 0

**Table 2.** The Percentage of Mutagenic Index (MI) and Damaged Cells Treated by Dillsun Herbal Medicine at Different Doses and their Comparison With Positive Control (Sodium Dichromate) at 60 and 120 Minutes After Exposure Which Indicates Significant Differences P < 0.001 a

Substance	MI	Damaged Cells (Score 2 + Score 3)
**Sodium Dichromate**		
**Dillsun 0, mg/mL**	53.5 ± 1.38	75.5 ± 1.67
1 h	1.6 ± 0.51	0.5 ± 0.57
2 h	1.7 ± 0.34	1 ± 0.65
**Dillsun 0.2, mg/mL**		
1 h	1.6 ± 0. 51	0.5 ± 0.5
2 h	1.7 ± 0.34	1 ± 0.65
**Dillsun 1, mg/mL**		
1 h	2.0 ± 0.4	1 ± 1.09
2 h	2.3 ± 0.4	1.5 ± 0.57
**Dillsun 2.5, mg/mL**		
1 h	2.6 ± 0.46	1.5 ± 1.09
2 h	3.0 ± 0.23	2 ± 1.09
**Dillsun 5, mg/mL**		
1 h	3.0 ± 0.46	2 ± 0.65
2 h	3.5 ± 0.23	3 ± 0.57
**Dillsun 10, mg/mL**		
1 h	3.75 ± 0.46	3 ± 0.93
2 h	4.0 ± 0.46	3.5 ± 1.51

^a^The Data are Expressed as the Mean ± SE

**Figure 1.**
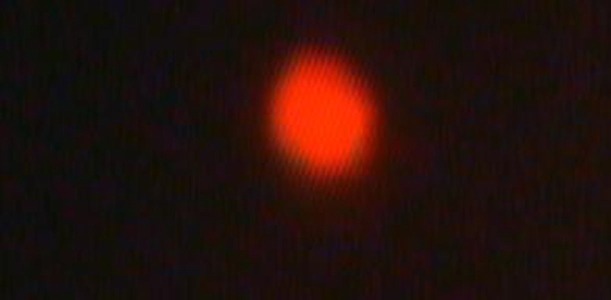
Microscopic Photograph 1 Score 0

**Figure 2.**
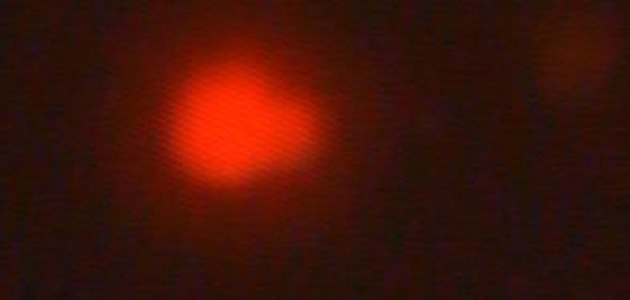
Microscopic Photograph 2 Score 1

**Figure 3.**
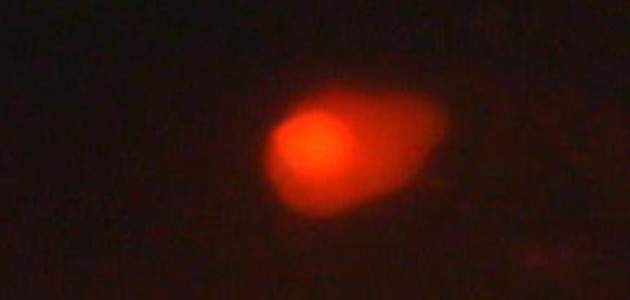
Microscopic Photograph 3 Score 2

**Figure 4.**
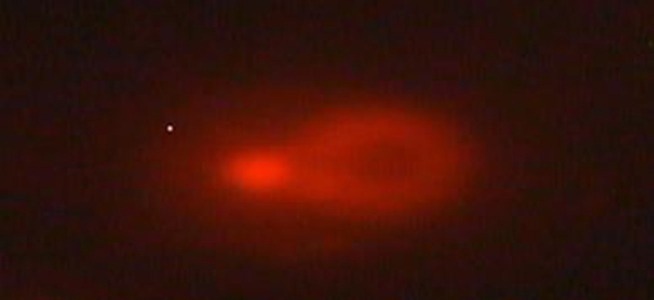
Microscopic Photograph 4 score 3

Comet lengths with scoring patterns, ranging from 0 to 3 is shown by microscopic images of Figures 1, 2, 3 and 4. Comets from the broken end, free of negative DNA molecules were moving in the electric field towards the anode. This method presented direct determination of the amount of DNA damage in single cells and the amount of DNA damages evaluated from the length of DNA migration. As expected, positive and negative controls compared with the test group showed a statistically significant difference. The results of the study indicated that the mutagenicity index of the positive control was 53.5%, the mutagenicity index for the lower dose of Dillsun after 60 and 120 minutes were 1.6% and 1.7% respectively, and the mutagenicity index of a higher dose of the Dillsun after 60 and 120 minutes were 3.75% and 4% respectively.

## 5. Discussion

Herbal medicine and natural substances as home remedies are widely used in developed and developing countries. These materials are used in the pharmaceutical industry and they are a significant ratio of the drug market. Therefore, it is necessary to do tests to evaluate their quality. Some herbs have become popular during the years, but there is no knowledge about the safety and effectiveness of herbal medicine and their usage in physicians, and some evidence demonstrate that these herbs can be dangerous. Standardization of herbals is necessary because they are used in herbal products to treat various acute and chronic diseases (7). In this study we used a comet assay because of its speed, simplicity and low cost and the small number of cells required. This test is very sensitive, rapid and flexible (8). Morkunas showed that oil of dill seed has no genotoxicity effects in mouse bone marrow (20). Hosseinzadeh showed that dill seed extracts have effects on secretion and ulcer induced by HCl and ethanol in the stomach (21). Thukham-mee et al. also showed that this substance can elevate acetylcholine and decrease oxidative stress while used at a low dose and for this reason dill can be effective on the nervous system and early phase of Alzheimer (22). The results of this study not only confirmed the results of most other studies yet it also showed that high doses of dillsun do not cause significant damage to DNA. But lazutka J.R et al. in 2000 showed that essential oil from dillsun herb was very active in SCE (sister chromatid exchange) and it included CA (chromosome aberrations). This herb includes a somatic mutation in *D. melanogaster*. They demonstrated that oil from dillsun seeds included SCE and CA in human lymphocytes, but it was inactive in SMART (somatic mutation and recombination test). They said that their unpublished study showed oil from dillsun seeds was very toxic for *Salmonella typhimurum* but none included mutation. In their study, they indicated that oil from dillsun was very cytotoxic for human lymphocytes and they were able to induce chromosome aberrations at low toxicity levels (23). Herbal medicines that have antioxidant properties can protect the cells against genotoxicity agents and prevent mutagenicity. In conclusion, the results showed that Dillsun has no mutagenic effects when compared to the positive control. Although by increasing the Dillsun dose, the DNA damage increased but this increase was not significant. Based on the other studies genotoxicity and toxicity of herbal medicine maybe very different, and it depends on the manufacturer, biogeographical conditions and (23) may vary during different seasons (20). So every product of every manufacturer must be evaluated for toxicity and genotoxicity.
